# Day-to-Day Fluctuation in Micronutrient Content in Human Milk Relative to Maternal Diet

**DOI:** 10.3390/nu16213727

**Published:** 2024-10-31

**Authors:** Noelia Ureta-Velasco, Adriana Montealegre-Pomar, Kristin Keller, Diana Escuder-Vieco, José C. E. Serrano, Nadia Raquel García-Lara, Carmen R. Pallás-Alonso

**Affiliations:** 1 Department of Neonatology, 12 de Octubre University Hospital, 28041 Madrid, Spain; nadiaraquelg.nrgl@gmail.com (N.R.G.-L.); kpallas.hdoc@gmail.com (C.R.P.-A.); 2 Research Institute i+12, 12 de Octubre University Hospital, 28041 Madrid, Spain; biol.kristin.keller@gmail.com (K.K.); diana.e.vieco@gmail.com (D.E.-V.); 3 Faculty of Medicine, Complutense University of Madrid, 28040 Madrid, Spain; 4 Epidemiología Clínica, Hospital Universitario San Ignacio, Pontificia Universidad Javeriana, Bogotá 110231, Colombia; montealegrepomar@gmail.com; 5 “Aladina-MGU”—Regional Human Milk Bank, 12 de Octubre University Hospital, 28041 Madrid, Spain; 6 Department of Experimental Medicine, Faculty of Medicine, University of Lleida, 25008 Lleida, Spain; jceserrano@mex.udl.cat

**Keywords:** breast milk, lactating women, generalized estimating equations, intake, vitamins, minerals, riboflavin, pyridoxal, vitamin D, selenium

## Abstract

Background/Objectives: The impact of daily variations in habitual maternal intake on human milk (HM) composition has been poorly studied. We aimed to investigate the associations between day-to-day fluctuation in the micronutrient concentration in HM and daily maternal diet. Methods: To this end, an observational longitudinal study was carried out. A total of 143 lactating women each compiled a five-day weighed dietary record and concomitantly expressed milk for four successive days. Maternal daily intakes of micronutrients and food groups were analyzed. Free thiamine, free riboflavin, nicotinamide, pantothenic acid, pyridoxal, folic acid, cobalamin, ascorbic and dehydroascorbic acids, retinol, alpha- and gamma-tocopherol, cholecalciferol, and calcidiol in HM were determined for each of the four investigated days. A longitudinal data analysis with generalized estimating equation models was performed. Results: For each daily serving of meat, fish, and eggs, total vitamin D_3_ levels increased by 243.2 pg/mL (*p* = 0.027) and selenium levels increased by 0.16 ppb (*p* = 0.031) in HM. For every 1 mg/day of riboflavin supplementation, free riboflavin levels in HM increased by 28.6 mcg/L (*p* = 0.019). Pyridoxal levels in HM increased by 6.3 mcg/L per 1 mg/day of vitamin B_6_ supplementation (*p* = 0.001), and by 2 mcg/L per daily fruit ration (*p* = 0.016). Conclusions: In conclusion, we were able to quantify the impact of the usual day-to-day variations in the diet and in the intake of commonly used supplements on the fluctuation of vitamin D, free riboflavin, pyridoxal, and selenium concentration in the milk of lactating women.

## 1. Introduction

Human milk (HM) is a dynamic fluid that changes depending on multiple factors, with maternal diet being one of the most relevant [1,2,3]. However, the effect of maternal diet on the composition of HM is not the same for all nutrients and in all circumstances [3,4].

A recently published systematic review [5] found strong evidence for the influence of maternal intake of iodine; selenium; vitamins A, E, and K; and the fatty acids docosahexaenoic acid (DHA) and eicosapentaenoic acid on the concentration of these nutrients in HM. Weaker evidence of the impact of maternal intake of linolenic acid, group B vitamins, and vitamins C and D on their concentration in milk was found in the same systematic review [5]. However, it is notable that the investigation of micronutrients is much more limited when compared to that of fatty acids [5,6]. Furthermore, as evidenced in the above-mentioned systematic review, most of the studies evaluating micronutrients were experimental and often involved interventions with supplement doses above the recommended daily intakes, while observational studies were in the minority [5].

In this regard, it is likely that in healthy women, the levels of some nutrients in HM, such as fat-soluble vitamins, vitamin B_1_, and vitamin C, respond only to high doses of supplementation and not to lower doses of supplementation or to variations within the habitual diet [4,5,7].

To our knowledge, the day-to-day changes in milk micronutrient composition attributed to daily fluctuations in usual diet and/or micronutrient intake in lactating women under non-experimental conditions and at a group level rather than individually have not yet been evaluated. However, this approach could be useful for better understanding the behavior of HM in response to dietary variations under real-life conditions. Such information could provide valuable support for establishing dietary recommendations for lactating women at the population level or in specific settings such as human milk banks.

Hence, our objective was to evaluate the association between variations in maternal dietary and supplementation intake and variations in HM micronutrient composition over four consecutive days.

## 2. Materials and Methods

### 2.1. Study Design and Participants

This observational longitudinal study was carried out at the Aladina MGU Regional Human Milk Bank (RHMB) at the “12 de Octubre” University Hospital in Madrid, Spain. A group of lactating mothers was recruited from August 2017 to February 2020, comprising (1) HM donors from the RHMB, (2) lactating mothers of very preterm infants (i.e., ≤32 weeks of gestational age (GA)-admitted at the time of the study to the Neonatology Service of the “12 de Octubre” University Hospital), and (3) lactating mothers with vegetarian or vegan diet.

The lactation time had to be at least 3 weeks, so that all of the milk samples analyzed corresponded to mature milk.

Exclusion criteria were malabsorptive intestinal disease, the use of drugs that could modify appetite, nutrient absorption or metabolism (antineoplastic drugs, anorectics, anabolic agents, diuretics, etc.), and idiomatic barriers.

The study was conducted in accordance with the Declaration of Helsinki and was approved by the Clinical Research Ethics Committee of the “12 de Octubre” University Hospital (protocol code 15/269). All participants provided written and informed consent.

### 2.2. Study Protocol

**Figure 1:**
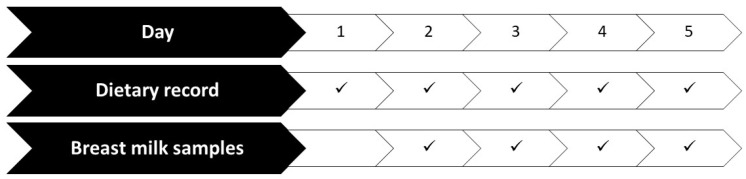
Protocol for completion of the dietary record and collection of milk samples over time.

At the beginning of the study, socio-demographic, health, and anthropometric data were collected from the participants and their breastfed children. Weight and height of the recruited lactating women were also measured, and their body mass indexes (BMIs) were calculated (electronic medical scale with BMI function Model 799 with measuring rod Model 220, CE approved—Class III, brand Seca^®^, Hamburg, Germany). Subsequently, they had to fill in a weighted dietary record for 5 consecutive days, including a holiday. Food intake and micronutrient supplementation were carefully reported. Detailed information on the recording method is described elsewhere [8]. Simultaneously, they collected milk for four consecutive days (from the second to the fifth day of the dietary record) (Figure 1).

Milk donors and lactating mothers with a vegetarian or vegan diet followed the same milk collection protocol: both groups of women collected at least one milk sample per day, without changing their milk expression routine and, therefore, without following a fixed schedule. The only requirement was to collect a complete milk expression. From each milk expression, after gently shaking, they collected 25 mL of milk with a sterile syringe, placing it in a sterile transparent glass bottle. The identification code as well as the date, time, and volume of the milk expression was labeled in the bottle before the sample was frozen to −20 °C. Mothers of very preterm infants admitted to our neonatal unit performed a 24 h milk expression. They collected a sample from each of the milk extractions carried out during the day to obtain a total volume of approximately 25 mL for each day. The rest of the procedure was the same as described above.

The five-day dietary record and milk samples were submitted to the RHMB within 15 days of study completion. The milk samples were transported to the RHMB in a portable cooler with cold accumulators. At RHMB, milk was thawed and, if available, milk samples from the same day were mixed, while milk from different days was not mixed, as the HM micronutrient composition of each day was analyzed individually. Twenty aliquots of 1 mL of the milk from each day were obtained. Each milk aliquot was labeled with the identification code and the day of the study to which it belonged. Subsequently, milk aliquots were kept frozen at −80 °C in the RHMB until they were sent for analysis.

### 2.3. Dietary Study

The weighed five-day dietary record was analyzed using DIAL-Software^®^ (DIAL.EXE version 3, February 2014, Alce Ingeniería, Madrid, Spain), and the nutrient intake for each day as well as the daily intake of each of the following food groups were obtained: (1) fruit; (2) vegetables and greens; (3) dairy; (4) grains, legumes, and nuts; and (5) eggs, meat, and fish. The daily intake from pharmacological supplements of each of the micronutrients investigated was also determined.

### 2.4. Micronutrient Analysis in HM

Micronutrient analyses in milk were performed by the NUTREN-Nutrigenomics Group of the Department of Experimental Medicine at the University of Lleida, Spain.

The following vitamins and minerals were analyzed in milk: free thiamine, free riboflavin, nicotinamide, pantothenic acid, pyridoxal, folic acid, cobalamin, ascorbic acid, dehydroascorbic acid, retinol, vitamin D_3_, 25(OH)D_3_, α-tocopherol, γ-tocopherol, iodine, calcium, phosphorous, and selenium. Table 1 shows the analytical techniques employed to determine each of the micronutrients studied. A full description of the laboratory studies is available in a previous manuscript [9].

### 2.5. Statistics

Statistical analyses were performed using the STATA 14 program.

Categorical variables were represented by the absolute and relative frequencies, whereas quantitative variables were described using medians and interquartile range or means, standard error, and standard deviation. Normality was determined by applying the Shapiro–Wilk test.

Longitudinal analyses were conducted to examine the possible association between daily changes in micronutrient levels in HM over a span of 4 days and the day-to-day intake of the equivalent micronutrients and supplements as well as different food groups. Hence, generalized estimating equations (GEE) models were employed using the vitamin and mineral concentration in HM as a dependent variable and the daily intake levels of different food groups, micronutrients, and supplements as independent variables. In this sense, the obtained results will inform about how the day-to-day fluctuations in the average micronutrient content in the HM of the investigated lactating mothers are related to the day-to-day fluctuations in their average micronutrient, supplement, and food group intake. A *p*-value of <0.05 was considered statistically significant.

## 3. Results

A total of 146 lactating women were recruited, of whom 3 dropped out of the study early, resulting in 143 women completing the study. Of the 143 women who completed the study, 113 were milk donors, 15 were mothers of very preterm infants admitted to the neonatal service, and 20 were vegetarians or vegans. The groups were not mutually exclusive. Thus, three of the donors were also mothers of very preterm infants admitted to the neonatal service, and another two were also ovo-lacto-vegetarians. In total, the study included 123 omnivores, 9 vegetarians (5 ovo-vegetarians and 4 ovo-lacto-vegetarians), and 11 vegans. Regarding the breastfed infants, at the time of the study, there were 17 preterm infants not admitted to the neonatal service (gestational age from 22^+6^ weeks to 36^+4^ weeks; from 38^+5^ weeks of postmenstrual age to 19.2 months of corrected age) and 19 very preterm infants admitted at the time of the study to the neonatal service (gestational age range from 23^+5^ weeks to 30^+3^ weeks; postmenstrual age range from 27^+4^ weeks to 41^+1^ weeks).

**Figure 2:**
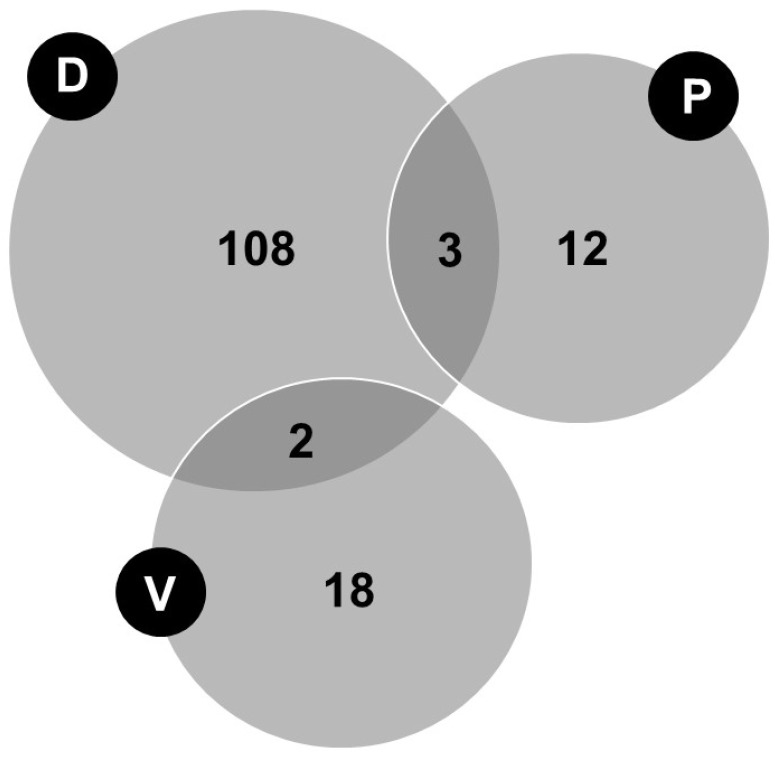
Distribution of participants (*n* = 143) by group. Abbreviations: D, human milk donors; P, mothers of preterm infants less than 32 weeks gestational age admitted to the neonatal service; V, vegetarian or vegan mothers. Number of total human milk donors = 113 (108 + 2 + 3). Number of total mothers of preterm infants less than 32 weeks of gestational age admitted to the neonatal service = 15 (12 + 3). Number of total vegetarian or vegan mothers = 20 (18 + 2).

Figure 2 shows the distribution of the participants by group.

### 3.1. Characteristics of the Studied Population

Results from the socio-demographic, health, and anthropometric data are presented in Table 2, Table 3, Table 4, Table 5 and Table 6. Furthermore, Table 7 shows the average vitamin and mineral concentration in human milk of lactating women collected during four successive days. In terms of nutrient supplementation reported in the socio-demographic and health survey, 141 of 143 lactating women (98.6%) declared using pharmacological micronutrient supplements during pregnancy, and 129 of 143 (90.2%) during lactation. Most participants took folic acid (*n* = 138 (96.5%)), vitamin B_12_ (*n* = 135 (94.4%)), and iodine (*n* = 136 (95.1%)) supplements during pregnancy.

### 3.2. Generalized Estimating Equations

Table 8 shows the association between day-to-day fluctuations in the average micronutrient content in HM across the entire sample of lactating women and the day-to-day fluctuations in their average food and supplement intake according to the dietary record.

For some of the micronutrients studied, the GEE analyses revealed some positive associations between lactating mothers’ intake and their concentration in milk over the days studied.

Regarding vitamins, (1) for each daily serving of meat, fish, and eggs, total vitamin D_3_ levels in HM increased by 243.2 pg/mL (*p* = 0.027); (2) for every 1 mg/day of riboflavin supplementation, free riboflavin levels in HM increased by 28.6 mcg/L (*p* = 0.019); and (3) regarding vitamin B_6_, pyridoxal levels in HM increased by 6.3 mcg/L per 1 mg/day of vitamin B_6_ supplementation (*p* = 0.001), and by 2 mcg/L per daily fruit ration (*p* = 0.016), according to the adjusted coefficients of the model.

Concerning minerals, for each daily serving of meat, fish, and eggs, the daily selenium levels in HM increased by approximately 0.16 ppb (*p* = 0.031).

No associations were found for the rest of the micronutrients studied (free thiamine, nicotinamide, pantothenic acid, folic acid, cobalamin, ascorbic acid, dehydroascorbic acid, retinol, α-tocopherol, γ-tocopherol, iodine, calcium, and phosphorous).

## 4. Discussion

To the best of our knowledge, this is the first study to longitudinally assess the effect of daily variations in the habitual diet and supplementation on daily variations in HM micronutrients. A positive association between the intake of daily servings of meat, fish, and eggs and the concentration of total vitamin D and selenium in HM was found. Likewise, the intake of vitamin B_2_ and B_6_ supplements contained in multi-nutrient supplements designed for lactating women in Spain was related to an increase in the content of free riboflavin and pyridoxal in HM. In addition, a higher daily intake of fruit was associated with an increased pyridoxal content in HM. The sample included healthy lactating women with different dietary patterns, gestational ages, and a wide range of lactation durations. Compared to other investigations regarding changes over time in HM micronutrient concentration, our study is an observational study conducted under real-life conditions. In this sense, no intervention or modification of diet or regular intake of micronutrient supplements was made. A detailed study of the diet was carried out through a weighted dietary record compiled for 5 days in a row. Concomitantly, women expressed milk for 4 consecutive days, and the micronutrient content was analyzed on each of the days studied.

The associations found in our study are consistent with the limited knowledge available from cross-sectional and experimental longitudinal studies.

In the current literature, vitamin B_2_ concentration in HM has been found to be associated with maternal vitamin B_2_ supplementation and intake [4,5,7,30,31,32]. In our study, we detected an association between the daily variation of the vitamin B_2_ concentration in HM and the daily vitamin B_2_ supplement intake. Likewise, the association of the daily variation in the concentration of vitamin B_6_ in HM with the day-to-day fluctuation of maternal vitamin B_6_ supplement intake found is in line with the results described in various systematic reviews [5,7]. However, there are studies that did not observe the association between maternal vitamin B_6_ intake and its concentration in HM [13,32]. Moreover, in our study, the daily variation in HM vitamin B_6_ concentration also showed an association with the number of daily fruit servings. This is consistent, as fruit is a good source of vitamin B_6_ [33].

Concerning selenium, the levels of this mineral are usually high in eggs, meat, poultry, fish, and seafood [34]. Indeed, a previous study conducted in Italy [35] found a positive association between maternal egg intake during gestation and fresh fish intake during lactation and the selenium content in HM. A recent systematic review concluded that there is a strong response of selenium content in milk to maternal selenium intake [5]. A transversal study in Galicia, a region of Spain, showed a positive correlation between adherence to the Mediterranean diet of lactating women and the concentration of selenium in their milk [36]. Regarding vitamin D, there is some evidence of the response of vitamin D in HM to the mother’s diet [4,5]. Eggs, meat, and fish are also good dietary sources of vitamin D, especially eggs, tuna, herring, and sardines [37]. However, concerns about the mercury content of fish can lead to restrictive fish consumption behaviors by pregnant and lactating women. Nevertheless, in previous studies, no significant relationship was found between mercury levels in HM and maternal consumption of fish [38,39]. Additionally, oily fish, such as salmon, sardines, mackerel, trout, anchovy, and herring, are valuable foods for breastfeeding mothers as they are a good source of selenium, vitamin D, and also omega-3, while being lower in mercury than other species [40,41].

On the other hand, we were not able to demonstrate the effect of habitual changes in mothers’ diets on the content in the milk of other micronutrients such as vitamin A, E, B1, C, and iodine. However, the concentration of all these nutrients in HM has been shown to be influenced by dietary or supplemental intake in some studies [3,5,7,8,31]. One possible explanation is that small daily variations in the usual maternal diet or low-dose supplementation of these nutrients are probably not sufficient to influence daily milk concentration in well-nourished women. In fact, in a systematic review, higher doses of supplementation showed greater effects [7].

Some limitations of our study need to be noted. First, we cannot rule out that we were not able to detect associations that truly exist, as we did not control for possible confounding factors such as circadian rhythm, or the time elapsed between intake and expression of milk [42]. In our study, we did not determine a schedule for milk sampling, but the women extracted their milk at the time that suited them best. Second, it is also feasible that a larger sample size would have yielded significant results for some of the micronutrients not associated with diet in the present study. Finally, although we measured HM nutrient concentration during four consecutive days, only one sample per day was obtained in the majority of the studied women.

In contrast, our study presents several noteworthy strengths. By analyzing the influence of basal diet and non-intervention supplementation on the composition of the HM in a wide cohort of healthy lactating women, we provide important insights into how common short-term changes in maternal intake affect HM micronutrient levels. This was achieved through a detailed study of lactating women’s diets concurrent with milk sampling. These findings suggest that even minor intake variations among healthy women can impact the nutritional composition of their milk, providing valuable information for designing population-level health education interventions.

The demonstration of the impact of daily dietary variations on the nutritional quality of HM underlines the importance of maternal nutrition during lactation. As the diet of lactating mothers is modifiable, prioritizing nutritional counseling becomes essential. In the setting of human milk banks and neonatal units, this insight could also drive initiatives to improve the intake by donors and mothers of preterm infants, thereby enhancing the nutritional quality of both donor human milk and mother’s own milk. Evidence underscores the crucial role of nutrient intake in the preterm infant on reducing short-term morbidity and improving long-term outcomes [43,44,45]. Furthermore, in developing countries, where animal-based foods may be scarce, integrating nutrient-dense therapeutic foods for lactating mothers can further enhance breastfeeding’s benefits.

## 5. Conclusions

In this observational longitudinal study, we were able to quantify the impact of the usual day-to-day variations in the diet and in the intake of commonly used supplements on the fluctuation in the concentration of vitamin D, free riboflavin, pyridoxal, and selenium in the milk of lactating women over the 4 days studied. This should encourage medical professionals to emphasize the importance of women’s nutrition during lactation and promote changes in their habits to improve their micronutrient intake and consequently their child’s health. This is particularly relevant in the context of human milk banks, neonatal units, and developing countries because of the vulnerability of the breastfed infants for whom the milk is intended.

## Figures and Tables

**Table 1:** Analytical techniques used for the determination of micronutrients in human milk.

Micronutrients	Analytical Technique
Free thiamine	UPLC tandem mass spectrometry
Free riboflavin	UPLC tandem mass spectrometry
Nicotinamide	UPLC tandem mass spectrometry
Pantothenic acid	UPLC tandem mass spectrometry
Pyridoxal	UPLC tandem mass spectrometry
Folic acid	UPLC tandem mass spectrometry
Cobalamin	Competitive immunoassay method
Ascorbic acid	HPLC-DAD
Retinol	HPLC with a fluorescence and UV detector
Vitamin D_3_	UPLC electrospray ionization/tandem mass spectrometry
25(OH)D_3_	UPLC electrospray ionization/tandem mass spectrometry
α-tocopherol	HPLC with a fluorescence and UV detector
γ-tocopherol	HPLC with a fluorescence and UV detector
Iodine	Inductively coupled plasma-mass spectrometry
Calcium	Inductively coupled plasma-mass spectrometry
Phosphorous	Inductively coupled plasma-mass spectrometry
Selenium	Inductively coupled plasma-mass spectrometry

Abbreviations: UPLC, ultra-performance liquid chromatography; HPLC, high-performance liquid chromatography; DAD, diode array detector; UV, ultraviolet.

**Table 2:** Lactating women characteristics (*n* = 143).

Characteristic	
Age (years)	35.61 (0.39) (4.67) *
Weight (kg)	61.20 (55.20, 70.60)
Height (cm)	163.85 (0.53) (6.39) *
Pre-pregnancy BMI (kg/m^2^)	22.16 (20.55, 24.92)
Pre-pregnancy BMI (kg/m^2^) category	
Underweight (<18.5)	6 (4.2%)
Normal (18.5–24.9)	103 (72.0%)
Overweight (25–29.9)	20 (14.0%)
Obese (≥30)	14 (9.8%)
Current BMI (kg/m^2^)	22.94 (21.12, 25.29)
Current BMI (kg/m^2^) category	
Underweight (<18.5)	5 (3.5%)
Normal (18.5–24.9)	101 (70.6%)
Overweight (25–29.9)	19 (13.3%)
Obese (≥30)	18 (12.6%)
Gestational weight gain (kg)	11.00 (8.00, 13.60)
Postpartum weight retention (kg)	1.00 (−0.60, 3.00)
Number of living children	
0 ^a^–1	81 (56.6%)
2	49 (34.3%)
≥3	13 (9.1%)
Country of origin: Spain	125 (87.4%)
Education level	
Secondary studies	6 (4.2%)
Technical studies	17 (11.9%)
University studies	120 (83.9%)
Currently working (Yes)	65 (45.5%)
Physical activity	
Sedentary	35 (24.5%)
Low activity	72 (50.3%)
Active/very active	36 (25.2%)
Tobacco consumption	
Prior to pregnancy (Yes)	28 (19.6%)
During pregnancy (Yes)	3 (2.1%)
Currently (Yes)	2 (1.4%)
Alcohol consumption	
Prior to pregnancy (Yes)	72 (50.3%)
During pregnancy (Yes)	2 (1.4%)
Currently (Yes)	7 (4.9%)
Season during the study	
Spring	30 (21.0%)
Summer	23 (16.1%)
Autumn	52 (36.4%)
Winter	38 (26.6%)

Quantitative variables following a normal distribution are described using the mean (standard error) (standard deviation), whereas the median (25th percentile, 75th percentile) is used for variables following a non-normal distribution. Qualitative variables are described by absolute and relative frequencies (%) of their categories. * Normal distribution. ^a^ One fetal death at 22^+6^ weeks of gestational age. Abbreviations: BMI, body mass index.

**Table 3:** Lactation characteristics.

Characteristic	*n*	
Duration of lactation of the previous child (months)	59 ^a^	
<3		0 (0.0%)
3–6		2 (3.4%)
6–12		12 (20.3%)
12–24		25 (42.4%)
>24		20 (33.9%)
Current breastfeeding time (months)	143	6.05 (4.27, 11.20)
Type of lactation	142 ^b^	
Exclusive		71 (50.0%)
Partial		71 (50.0%)
Sum of breastfeeding times plus daily milk pumping sessions	143	
<5		14 (9.8%)
5–10		87 (60.8%)
>10		39 (27.3%)
Missing data		3 (2.1%)
Tandem nursing * (Yes)	143	10 (7.0%)
Breastfeeding twins (Yes)	143	4 (2.8%)
Type of milk extraction **	143	
Manual		12 (8.4%)
Mechanical breast pump		15 (10.5%)
Simple electric breast pump		101 (70.6%)
Double electric breast pump		26 (18.2%)

The variable “current breastfeeding time” is expressed as a median (25th percentile, 75th percentile) because of its non-parametric distribution. Qualitative variables are described by absolute and relative frequencies (%). ^a^ Women with previous children. ^b^ Sample size decreases due to one fetal death. * Breastfeeding two or more children of different ages. ** Categories do not exclude each other.

**Table 4:** Baseline characteristics of the infants of the investigated lactating women.

Characteristic	*n*	
Gestational age (weeks)	143	39^+3^ (37^+3^, 40^+2^), 22^+6^–42^+3^
Boy	148 ^a^	72 (48.6%)
Birth weight (grams)	148 ^a^	3150 (2550, 3420), 450–4640
Birth weight percentile ^1^		
≤25		49 (33.1%)
25–75	148 ^a^	84 (56.8%)
≥75		15 (10.1%)
Age of infant (months)	146 ^b^	
0–6		73 (50.0%)
6–12		41 (28.1%)
12–50		32 (21.9%)
Current weight percentile of breastfed child ^2^	146 ^b^	
≤15		32 (21.9%)
15–85		94 (64.4%)
≥85		20 (13.5%)

Quantitative variables are shown as medians (25th percentile, 75th percentile) because of their non-parametric distribution. Range is displayed after the coma. Qualitative variables are described by absolute and relative frequencies (%) of their categories. ^a^ The sample size increases due to births of twins (*n* = 5). ^b^ Sample size decreases due to a fetal death and a postnatal death. ^1^ Olsen intrauterine growth curve [10]. ^2^ World Health Organization (WHO)’s child growth standards [11].

**Table 5:** Consumption of pharmacological micronutrient supplements during the dietary record in lactating women (*n* = 143).

Supplement	*n* (%) *	Daily Dose
Vitamin A, mcg	43 (30.1)	640.0 (400.0, 800.0)
Vitamin D, mcg	56 (39.2)	5.0 (3.0, 5.0)
Vitamin E, mg	52 (36.4)	11.2 (6.3, 12.0)
Vitamin C, mg	51 (35.7)	80.0 (48.0, 80.0)
Vitamin B_1_, thiamine, mg	51 (35.7)	1.1 (0.7, 1.1)
Vitamin B_2_, riboflavin, mg	51 (35.7)	1.4 (0.8, 1.4)
Vitamin B_3_, niacin, mg	51 (35.7)	15.6 (9.6, 16.0)
Vitamin B_5_, pantothenic, mg	51 (35.7)	5.7 (3.6, 6.0)
Vitamin B_6_, pyridoxine, mg	51 (35.7)	1.4 (0.8, 1.4)
Vitamin B_7_, biotin, mcg	51 (35.7)	50.0 (30.0, 50.0)
Vitamin B_9_, folic acid, mcg	72 (50.3)	290.0 (165.0, 400.0)
Vitamin B_12_, cobalamin, mcg	78 (54.5)	2.0 (1.6, 2.5)
Iodine, mcg	75 (52.4)	160.0 (120.0, 200.0)
Calcium, mg	38 (26.6)	160.0 (100.0, 200.0)
Iron, mg	54 (37.8)	14.0 (8.4, 17.7)
Zinc, mg	48 (33.6)	8.0 (5.3, 10.0)
Selenium, mcg	46 (32.2)	20.0 (11.5, 45.0)

Daily dose is expressed as median (25th percentile, 75th percentile) due to the non-parametric distribution of the variables. Qualitative variables are described by absolute and relative frequencies (%). * Number and percentage of lactating women taking supplements during the dietary record.

**Table 6:** Prevalence of inadequate intakes of micronutrients in lactating women (*n* = 143).

Nutrient	H-AR [12]	*n* (%)
Thiamine (B_1_), mg	1.2	11 (7.7)
Riboflavin (B_2_), mg	1.7	29 (20.3)
Niacin (B_3_), mg	13	0 (0.0)
Pantothenic acid (B_5_), mg	5.6	38 (26.6)
Pyridoxine (B_6_), mg	1.4	3 (2.1)
Biotin (B_7_), μg	36	52 (36.4)
Folate food + folic acid (B_9_), μg	380 (DFE)	50 (35.0)
Cobalamin (B_12_), μg	2.4	5 (3.5)
Vitamin C, mg	145	51 (35.7)
Vitamin A, μg RAE	1020	45 (31.5)
Vitamin D, μg	10	122 (85.3)
Vitamin E, mg	16	70 (49.0)
Iodine, μg	209	60 (42.0)
Calcium, mg	860 (19–30 y) 750 (31–50 y)	23 (16.1)
Phosphorous, mg	580	0 (0.0)
Selenium, μg	59	4 (2.8)

The number and percentage of lactating women with inadequate intakes of each nutrient (average nutrient intake during the 5 days of the dietary record below harmonized average requirements) are presented. Abbreviations: H-AR, Harmonized-Average Requirements; DFE, dietary folate equivalents; RAE, retinol activity equivalents; and y, years.

**Table 7:** Average vitamin and mineral concentration in human milk of lactating women collected during four successive days.

Nutrient ^1^	Lactating Women		Mature Milk Nutrient Concentration Reference
	*n* (o)	Concentration	
Free thiamin, B_1_ (mcg/L)	143 (570)	17.90 (9.08, 27.90)	Free thiamin 18.5 [13] Total thiamin 180 [14]
Free riboflavin, B_2_ (mcg/L)	143 (570)	40.38 (20.45, 97.70)	Free riboflavin 11.2 [13] Total riboflavin 364 [15]
Nicotinamide, B_3_ (mcg/L)	143 (570)	43.33 (26.23, 77.15)	Nicotinamide 275 [13] Total niacin 2100 [16]
Pantothenic acid, B_5_ (mcg/L)	143 (570)	2205.76 (39.62) (473.83)	2500 [17] 1304 [13]
Pyridoxal, B_6_ (mcg/L)	143 (570)	36.73 (24.78, 53.90)	Pyridoxal 96 [13] B6 130 [18]
Folic acid, B_9_ (mcg/L)	143 (570)	18.48 (14.25, 23.70)	
Cobalamin, B_12_	142 (565)		
pM		482.46 (448.34, 533.41)	
mcg/L		0.65 (0.61, 0.72)	0.5 [19]
Ascorbic acid (mg/dL)	142 (566)	4.10 (0.15) (1.84)	
Dehydroascorbic acid (mg/dL)	142 (566)	1.86 (1.15, 2.81)	
Vitamin C *	142 (566)		
mg/dL		6.40 (5.51, 7.16)	
mg/L		63.98 (55.07, 71.61)	35–90 [20]
Retinol	141 (561)		
mcg/dL		45.45 (27.29, 92.23)	
mcg/L		454.50 (272.88, 922.25)	530 [21]
Vitamin D_3_	143 (566)		
pg/mL		1132.95 (279.63, 4823.43)	
mcg/L		1.13 (0.28, 4.82)	0.25–2 [22]
25(OH)D_3_	143 (567)		
pg/mL		61.20 (27.60, 118.85)	
mcg/L		0.06 (0.03, 0.12)	
α-tocopherol	141 (561)		
mcg/dL		441.63 (362.09, 558.06)	
mg/L		4.42 (3.62, 5.58)	4.6 [23]
γ-tocopherol	141 (561)		
mcg/dL		51.25 (38.01, 69.83)	
mg/L		0.51 (0.38, 0.70)	0.45 [13]
Vitamin E (as TE) **	141 (561)		
mcg/dL		458.91 (379.28, 580.73)	
mg/L		4.59 (3.79, 5.81)	5.2 [13]
Iodine (mcg/L)	143 (570)	142.65 (92.93, 207.28)	50–100 [18] 100–200 [24,25]
Calcium (mg/L)	143 (570)	92.40 (61.25, 126.10)	200–300 [26]
Phosphorous (mg/L)	143 (570)	130.55 (2.18) (26.12)	120–140 [18,27]
Selenium (mcg/L)	143 (570)	11.03 (9.25, 13.08)	18 [28]

Quantitative variables following a normal distribution are described by the mean (standard error) (standard deviation), whereas the median (25th percentile, 75th percentile) is used for variables following a non-normal distribution. *n*: number of lactating women with data. o: total number of the samples (observations) corresponding to each of the four human milk collection days. ^1^ The units of our results were converted to the units of the reference values for comparability. * Vitamin C (mg/dL) = ascorbic acid (mg/dL) + dehydroascorbic acid (mg/dL). ** Vitamin E (TE) (mg) = α-tocopherol (mg) + 0.25 × γ-tocopherol (mg) [29]. Abbreviations: TE, tocopherol equivalent.

**Table 8:** Longitudinal analysis of the relationship between changes in micronutrient content in human milk and fluctuation in mothers’ nutrient, supplement, and food group intake according to the 5-day dietary record (GEE models).

Micronutrients in HM	Associated Variables	Coef	SE	z	*p* > |z|	95% CI
Vitamin D_3_ + 25(OH)D_3_, pg/mL Mothers = 143 Wald chi^2^(1) = 4.88 *p* > chi^2^ = 0.027	Meat, Fish, and eggs servings/day	243.203	110.057	2.21	0.027	[27.494, 458.911]
Free riboflavin, mcg/L Mothers = 51 Wald chi^2^(2) = 39.56 *p* > chi^2^ = <0.001	B_2_ intake * mg/day	7.312	11.541	0.63	0.526	[−15.308, 29.931]
	B_2_ supplement mg/day	28.642	12.214	2.35	0.019	[4.703, 52.581]
Pyridoxal, mcg/L Mothers = 51 Wald chi^2^(2) = 17.08 *p* > chi^2^ = <0.001	B_6_ supplement mg/day	6.284	1.885	3.33	0.001	[2.589, 9.978]
	Fruit servings/day	2.006	0.831	2.41	0.016	[0.377, 3.634]
Selenium, ppb Mothers = 143 Wald chi^2^(1) = 4.67 *p* > chi^2^ = 0.031	Meat, Fish, and eggs servings/day	0.158	0.073	2.16	0.031	[0.015, 0.301]

In free riboflavin and pyridoxal models, 51 mothers registered intake of vitamin B_1_ and vitamin B_6_ supplements. * Control variable. Abbreviation: coef, coefficient; SE, standard error; ppb, parts per billion.

## Data Availability

The data presented in this study are available on request from the corresponding author. The data are not publicly available due to privacy issues.
